# A new subspecies and a new synonym of the genus *Coladenia* (Hesperiidae, Pyrginae) from China

**DOI:** 10.3897/zookeys.518.10011

**Published:** 2015-08-25

**Authors:** Guo-Xi Xue, Yutaka Inayoshi, Hua-Lin Hu, Meng Li, Ying-Dang Ren

**Affiliations:** 1 School of Food and Bioengineering, Zhengzhou University of Light Industry, No. 5 Dongfeng Road, Zhengzhou, Henan, 450002, China; 2 Sritana Condominium 2, 96/173, Huay Kaew Rd., T. Suthep, A. Muang, Chiang Mai, 50200, Thailand; 3 Jiulianshan National Nature Reserve, Longnan, Ganzhou, Jiangxi Province 341000, China; 4 Institution of Plant Protection, Henan Academy of Agricultural Sciences, No. 116 Huayuan Road, Zhengzhou, Henan, 450002, China

**Keywords:** Taxonomy, wing pattern, genitalia, fauna, distribution, *Coladenia buchananii separafasciata*, *Coladenia neomaeniata*, *Coladenia maeniata*

## Abstract

The second subspecies of *Coladenia buchananii* (de Nicéville, 1889), viz. *Coladenia buchananii separafasciata* Xue, Inayoshi & Hu, **ssp. n.**, is discovered from south Jiangxi Province and west Fujian Province, southeast China. External and genital characters of both male and female of this new subspecies are illustrated and described. *Coladenia neomaeniata* Fan & Wang, 2006, **syn. n.** is proposed to be a junior synonym of *Coladenia maeniata* Oberthür, 1896, and the distribution of this species is briefly discussed.

## Introduction

*Coladenia buchananii* (de Nicéville, 1889) was described based upon a single female collected in north Myanmar. It is distributed in Myanmar (Evans 1949), Thailand (Ek-Amnuay 2006) and Laos (Osada et al. 1999), and was recorded for the first time from northwest Yunnan of China by Huang (2003) with a brief note on its habit.

During our study of the butterfly fauna in Jiulianshan National Nature Reserve, south Jiangxi Province, we captured two male skippers, which possess male genitalia nearly identical to those of *Coladenia buchananii*, but their wing patterns are conspicuously different from the latter. Considering the geographic gap between south Jiangxi and the range of *Coladenia buchananii*, we believe that the two specimens represent a new subspecies of *Coladenia buchananii*. Additionally, while sorting the butterfly collection in the Institute of Zoology, Chinese Academy of Sciences, the first author found a female specimen collected from west Fujian Province more than 20 years ago, and it bears almost the same wing markings as the two males from south Jiangxi. Since both sexes of all the known species in the genus *Coladenia* always have similar appearance, this female should undoubtedly belong to the new subspecies from Jiangxi. Thus, we describe this new subspecies in the present paper.

Besides, *Coladenia neomaeniata* Fan & Wang, 2006 is treated as a junior synonym of *Coladenia maeniata* Oberthür, 1896, because they have identical male genitalia, and according to the existing distributional data, their external differences do not show a subspecific division and can only be considered as intraspecific individual variation. The range of this species is clarified based on information from literature and specimens.

## Materials and methods

The following specimens of *Coladenia buchananii buchananii* (de Nicéville, 1889) were examined and compared with the new subspecies: Thailand: 1 male, Chiang Mai, Doi Suthep, 4 Aprial 1983; 2 males, *ditto*, 3 Aprial 1987; 2 males, *ditto*, 7 Aprial 1987; 1 male, *ditto*, 16 March 1988; 2 males, *ditto*, 12 Aprial 1988; 1 male, *ditto*, 26 March 1992; 1 male, Tak, Umphang, Ya Mo Kwi, 17 March 1994; 1 male, Lampang, Mae Pam, 10 Aprial 2014. All these specimens are in the private collection of the junior author of the present paper.

Specimens of *Coladenia maeniata* Oberthür, 1896 listed as follows were examined and compared with *Coladenia neomaeniata* Fan & Wang, 2006. China: Yunnan: 3 males, Gongshan, Bingzhongluo, 16 May 2011; 3 males, Deqin, 3000 m, 18 June 2014. All these specimens were collected by Mr. Chun-Hao Wang, Beijing, and are preserved in his private collection.

The Comstock-Needham venation system was used in this paper. The terminology of genitalia mainly follows that of Shirôzu (1960).

The holotype and the male paratype of the new subspecies are deposited in School of Food and Bioengineering, Zhengzhou University of Light Industry. The female paratype is kept in the Institute of Zoology (IOZ), Chinese Academy of Sciences.

## Results

### Coladenia buchananii separafasciata

Taxon classificationAnimaliaLepidopteraHesperiidae

Xue, Inayoshi & Hu ssp. n.

http://zoobank.org/ECE754E0-3281-4EB4-8DC8-CC9961556F4D

Figs 1–3 , 4 , 5–12 , 13

#### Description.

**Figures 1–3.**
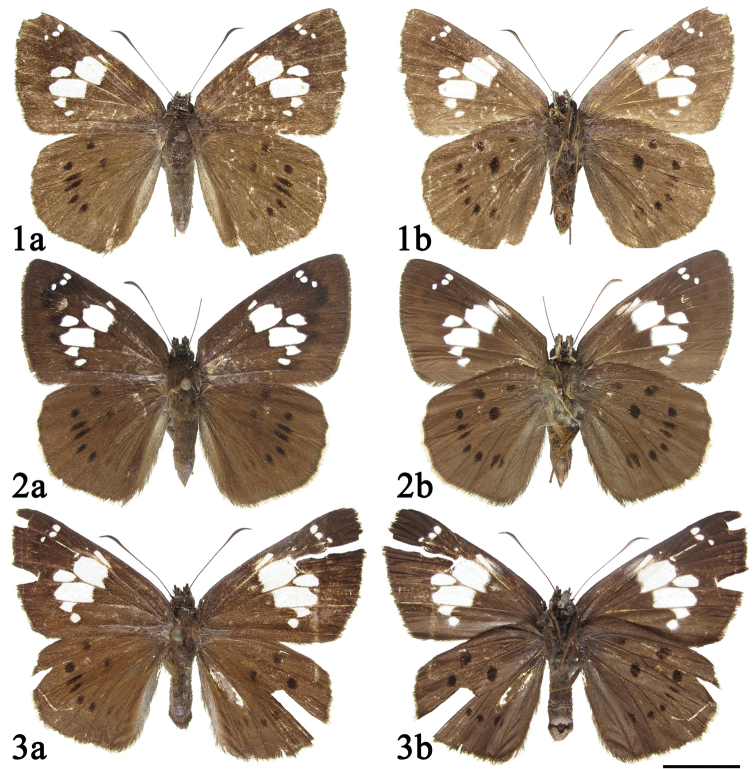
Adults of *Coladenia buchananii separafasciata* Xue, Inayoshi & Hu, ssp. n. **1–2** male **3** female **a** dorsal side **b** ventral side. Scale bar: 1 cm.

**Figure 4.**
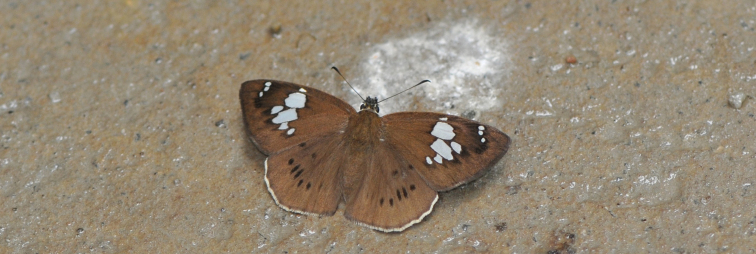
Male adult of *Coladenia buchananii separafasciata* Xue, Inayoshi & Hu, ssp. n. in the nature. Photo by Hua-Lin Hu at the type locality, 17 April 2013.

Male. (Figs 1–2, 4) Antennae: 12.5 mm in length, dorsal side shaft dark brown and club black, ventral side basal half dark brown and distal half covered with milky white scales, apiculus slender and sharply pointed. Labial palpi: second segment covered with white scales and black hairs, mixed with some black scales; third segment porrect, with a blunt point, much thicker than the shaft of antennae, dorsal side black, ventral side with white and black scales. Thorax and abdomen: dark brown, hind tibiae with a pale yellowish hair pencil. Forewing: 22.5 mm in length, dorsal side dark brown, spots white; apical spot in space r_4_ closer to the one in r_3_ than to that in r_5_; strip in space sc shorter than half length of widest portion of cell spot; cell spot big, rectangular, inner edge straight and outer upper angle concave; spot in space cu_1_ narrower and longer than cell spot, its outer lower angle protrudent, overlapping with cuniform spot in space m_3_; under spot in space cu_1_ there are two separated spots in space cu_2_, of which the lower one moved inwards; all apical and discal spots are accompanied by dark shadows. Ventral side of forewing, white spots repeat those on above, except there is a white strip in space c before the one in sc. Hindwing dark brown dorsally, basal area covered with hairs; discal area with a series of black spots from space sc+r_1_ to cu_2_, of which the two in spaces m_1_–m_2_ longer and looked like an equal sign, and the two in cu_2_ blurred; cell with an obscure black spot. Ventral side, all spots in discal series clearly present, base of space sc+r_1_ with black spot, cell spot distinct and bigger than all other spots. Cilia on forewing dark brown, mixed with milky white at end of the upper half of space cu_2_; cilia on hindwing milky white.

**Figures 5–12.**
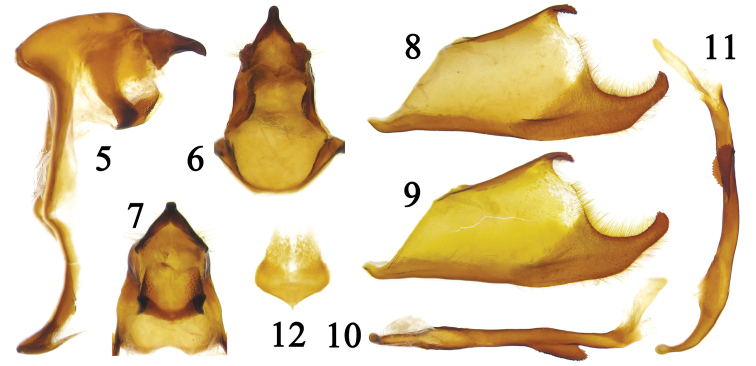
Male genitalia of *Coladenia buchananii separafasciata* Xue, Inayoshi & Hu, ssp. n. **5** ring, lateral view **6** uncus, dorsal view **7** gnathos, ventral view **8** right valva, inner view **9** left valva, outer view **10** aedeagus, dorsal view **11** aedeagus, lateral view **12** juxta.

Male genitalia (Figs 5–12). Tegumen produced forwards and dorsally humpy in lateral view. Uncus beak-like with a sharp point in lateral view, tapered and elongated to a short finger-like blunt tip in dorsal view; its base with an auriform process on each side. Gnathos arm-like in lateral view, connected on ventral side, its tip with tiny teeth. The upper half of the ring straight, and the lower half curved. Saccus very short. Valva broad, trapezoidal, with its distal portion widely bifid into two branches, of which the upper one short and curved downwards, decorated with small teeth, the lower one elongated and curved upwards, its tip with tiny teeth. Aedeagus a little shorter than ventral margin of valva; in lateral view, coecum penis slender and curved upwards, with a rounded head; subzonal sheath shorter than suprazonal sheath; left side of the middle of suprazonal sheath bear a leaflike sheet, its edge with sawteeth. Juxta cordiform.

Female (Fig. 3). Similar to male. Forewing 22.5 mm in length, strip in space sc longer than half length of widest portion of cell spot; end of abdomen with dense gray hairs.

**Figure 13.**
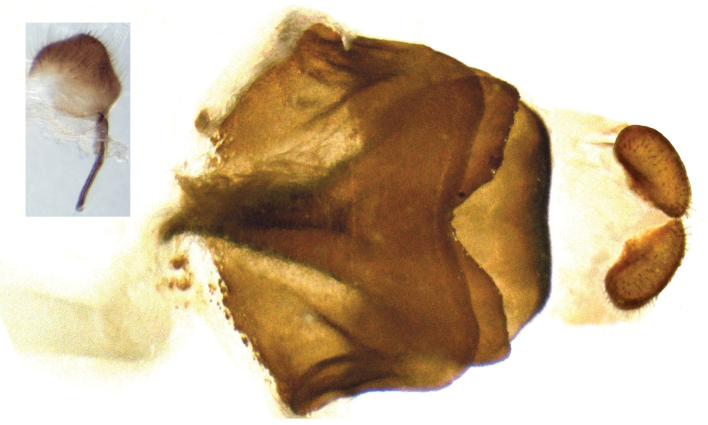
Female genitalia of *Coladenia buchananii separafasciata* Xue, Inayoshi & Hu, ssp. n., ventral view, with a close-up view of the apophyses posteriors.

Female genitalia (Fig. 13). Papillae anales reniform, covered with short hairs. Apophyses posteriors a little longer than papillae anales. Lamella postvaginalis wide, with flat edge. The middle of the edge of lamella antevaginalis widely V-shaped. Sternum of seventh segment of abdomen sclerotized into a solid plate, with its posterior edge shallowly coved. Ductus bursae and bursa copulatrix bursiform, membranous, without signum.

#### Type material.

Holotype: male, dry pinned, with genitalia preserved in glycerin. China: Jiangxi Province, Longnan County, Jiulianshan National Nature Reserve, Xiagongtang, 600 m, 7 May 2013, leg. Hua-Lin Hu. Paratypes: 1 male, *ditto*, 2 May 2013; 1 female, dry pinned, IOZ(E)1687887, with genitalia preserved in glycerin. China: Fujian Province, Jiangle County, Longqishan, Lishan, 650 m, 20 May 1991, leg. Hong-Xing Li.

#### Distribution.

China (S. Jiangxi, W. Fujian).

#### Difference with *Coladenia buchananii buchananii* (de Nicéville, 1889)

**Figures 14–15.**
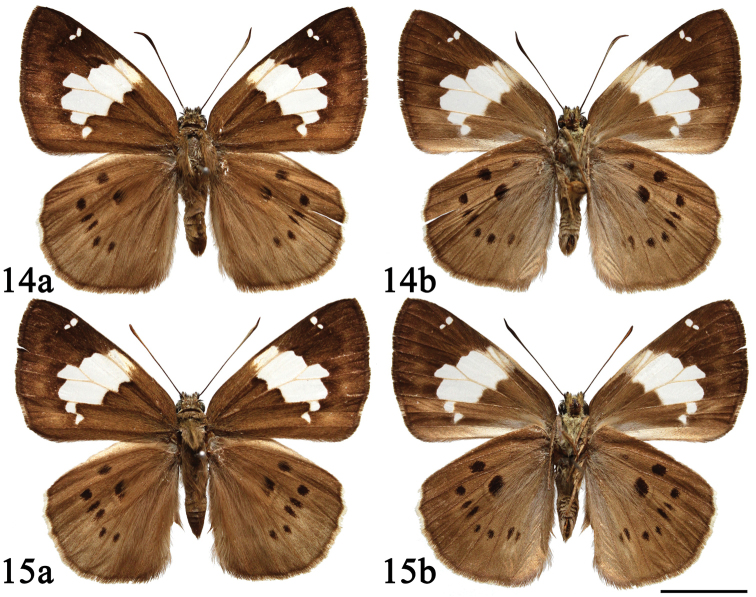
Male adults of *Coladenia buchananii buchananii* de Nicéville, 1889. **a** dorsal side **b** ventral side. Scale bar: 1 cm.

**Figures 16–23.**
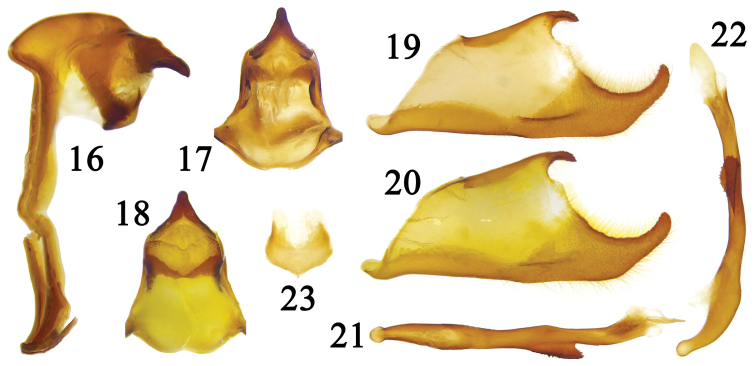
Male genitalia of *Coladenia buchananii buchananii* de Nicéville, 1889. **16** ring, lateral view **17** uncus, dorsal view **18** gnathos, ventral view **19** right valva, inner view **20** left valva, outer view **21** aedeagus, dorsal view **22** aedeagus, lateral view **23** juxta.

Eleven male specimens of *Coladenia buchananii buchananii* (de Nicéville, 1889) were collected from Thailand by the second author of this paper, two of them were dissected and illustrated herein (Figs 14–15, 16–23). According to these specimens and the images of female *Coladenia buchananii* in literature (de Nicéville 1889, Swinhoe 1912–1913, Ek-Amnuay 2006), the nominate subspecies is distinguishable from the new subspecies by the following combination of characters:

1. Ground color on both sides of the wings is paler.
2. On the dorsal side of forewing, the discal spots closely connected with each other and formed a wide band; the strips in space c and space sc combined into a wide bar, longer than half the length of the widest portion of cell spot. On the ventral side of forewing, the discal band reaches Costa.
3. Cilia on hindwing is milky white before the end of vein M_1_, and brown from the end of vein M_1_ to the tornus.
4. The base of uncus in male genitalia without auriform process.
5. The coecum penis of aedeagus is thicker and shorter, not conspicuously constricted before the head.

#### Bionomics.

Year round collecting indicates that this new subspecies is probably univoltine, only known from mid April to mid May, about one month later than the nominate subspecies which mainly appears from mid March to mid April. The two male types were captured at roadside, very near to a residential area in Jiulianshan National Nature Reserve.

#### Etymology.

The subspecific epithet is a combination of the prefix separa- and the Latin fasciata, referring to the broken band on forewing.

### Coladenia maeniata

Taxon classificationAnimaliaLepidopteraHesperiidae

Oberthür, 1896

Coladenia maeniata Oberthür, 1896: 42, pl. 9, fig. 164 (original description). Type locality: Maenia, Thibet [sic]; Elwes and Edwards 1897: 130 (diagnosis, distribution); Swinhoe 1912–1913: 71 (distribution); Evans 1949: 118 (description, distribution); Huang 2003: 68 (distribution); Fan and Wang 2006: 79, 80 (distribution, diagnosis).
Coladenia neomaeniata Fan & Wang, 2006: 79 (original description). Type locality: Weixi County, Yunnan Province. syn. n.

#### Notes.

**Figures 24–29.**
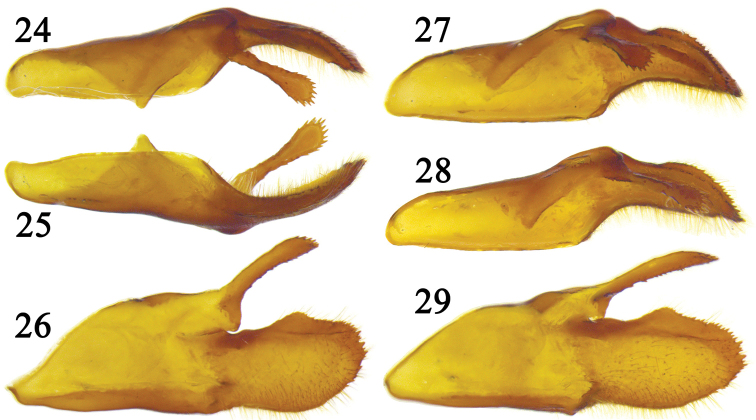
Right valva of the male genitalia of *Coladenia maeniata* Oberthür, 1896. **24** dorsal view **25** ventral view **26–29** inner side from different view angle.

**Figures 30–31.**
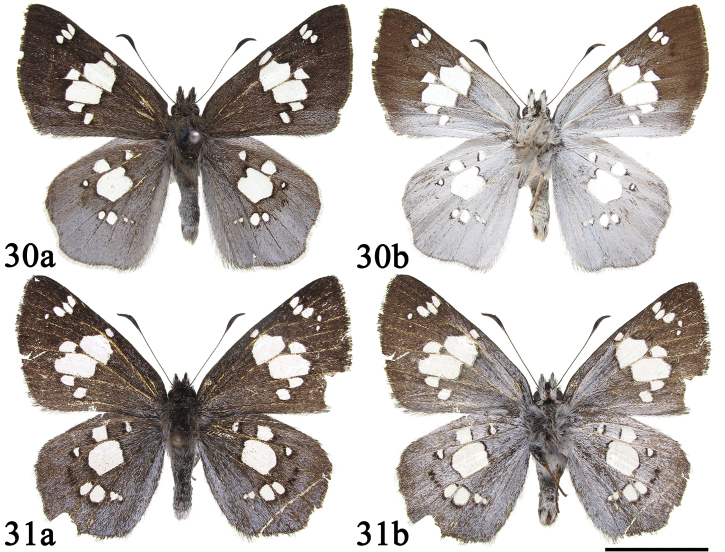
Male adults of *Coladenia maeniata* Oberthür, 1896. **a** dorsal side **b** ventral side. Scale bar: 1 cm.

According to Fan and Wang (2006), *Coladenia neomaeniata* is different from *Coladenia maeniata* in having the valva of the male genitalia conspicuously narrowed distally, and the inner edge of the spot in space sc+r_1_ on hindwing not in line with that of the cell spot. The first author of the present paper examined some specimens from northwest Yunnan which have the same wing pattern as *Coladenia neomaeniata*, and found that the valva of the male genitalia is bowl-like (Figs 24, 25), and its inner side looks different depending on the angle of view (Figs 26–29): from a certain angle to see, its distal part is narrowed as shown by figure 5 in Fan and Wang (2006) (Fig. 27), but from another view angle, it agrees with the figure provided by Evans (1949) (Fig. 26); besides, figure 6 in Fan and Wang (2006) is the dorsal view of the valva, not later view. Thus, the male genitalia of *Coladenia neomaeniata* is actually identical to that of *Coladenia maeniata*. Moreover, *Coladenia neomaeniata* is in the distributional range of *Coladenia maeniata* (Evans 1949, Fan and Wang 2006), so their difference in the position of the spot in sc+r_1_ on hindwing should be considered as intraspecific individual variation rather than subspecific differentiation. Such variation also appears in the shape of the spot in space sc on forewing which changes from a tiny dot to a long strip (Fan and Wang 2006, Oberthür 1896) (Fig. 30), and also in the spots in spaces m_1_ and m_2_ which may be fully developed (Fig. 31), vestigial (Oberthür 1896) or absent (Fan and Wang 2006). Therefore, *Coladenia neomaeniata* is treated as a junior synonym of *Coladenia maeniata* herein.

#### Remarks.

Fan and Wang (2006) recorded the distribution of *Coladenia maeniata* in southeast Xizang. We have not found any information from their work and other literature which shows this species is distributed in southeast Xizang, except that the type locality was recorded as Maenia, E. Tibet (Elwes and Edwards 1897). But the geographic range of Tibet in old entomological literature is much bigger than that of Xizang (= Tibet Autonomous Region) in nowadays. According to Elwes and Edwards (1897), Maenia is a place near Ta-tsien-lo, viz. Ta-tsien-lu, which is now known as Kangding area in west Sichuan Province, not in the range of Xizang. Then, the distribution of *Coladenia maeniata* should be clarified as northwest Yunnan and west Sichuan (Evans 1949).

## Supplementary Material

XML Treatment for Coladenia buchananii separafasciata

XML Treatment for Coladenia maeniata
